# Acquisition of Innate Inhibitor Resistance and Mammalian Pathogenicity During Egg Adaptation by the H9N2 Avian Influenza Virus

**DOI:** 10.3389/fmicb.2018.01939

**Published:** 2018-08-21

**Authors:** Chung-Young Lee, Se-Hee An, Jun-Gu Choi, Youn-Jeong Lee, Jae-Hong Kim, Hyuk-Joon Kwon

**Affiliations:** ^1^Laboratory of Avian Diseases, College of Veterinary Medicine, Seoul National University, Seoul, South Korea; ^2^Avian Disease Division, Animal and Plant Quarantine Agency, Gimcheon-si, South Korea; ^3^Research Institute for Veterinary Science, College of Veterinary Medicine, Seoul National University, Seoul, South Korea; ^4^Department of Farm Animal Medicine, College of Veterinary Medicine, Seoul National University, Seoul, South Korea; ^5^Farm Animal Clinical Training and Research Center, Institutes of Green-bio Science & Technology, Seoul National University, Gangwon-do, South Korea

**Keywords:** avian influenza virus, egg adaptation, hemagglutinin, neuraminidase, innate inhibitor, mammalian pathogenicity, H9N2

## Abstract

An H9N2 avian influenza A virus (AIV), A/chicken/Korea/01310/2001 (01310-CE20), was established after 20 passages of influenza A/chicken/Korea/01310/2001 (01310-CE2) virus through embryonated chicken eggs (ECEs). As a result of this process, the virus developed highly replicative and pathogenic traits within the ECEs through adaptive mutations in hemagglutinin (HA: T133N, V216G, and E439D) and neuraminidase (NA: 18-amino acid deletion and E54D). Here, we also established that 01310-CE20 acquired resistance to innate inhibitors present in the egg white during these passages. To investigate the role of egg-adapted mutations in resistance to innate inhibitors, we generated four PR8-derived recombinant viruses using various gene combinations of HA and NA from 01310-CE2 and 01310-CE20 (rH_2_N_2_, rH_2_N_20_, rH_20_N_2_, and rH_20_N_20_). As expected, rH_20_N_20_ showed significantly higher replication efficiency in MDCK cells and mouse lungs, and demonstrated greater pathogenicity in mice. In addition, rH_20_N_20_ showed higher resistance to innate inhibitors than the other viruses. By using a loss-of-function mutant and receptor-binding assay, we demonstrated that a T133N site directed mutation created an additional N-glycosite at position 133 in rH_20_N_20_. Further, this mutation played a crucial role in viral replication and resistance to innate inhibitors by modulating the binding affinities to avian-like and mammalian-like receptors on the host cells and inhibitors. Thus, egg-adapted HA and NA may exacerbate the mammalian pathogenicity of AIVs by defying host innate inhibitors as well as by increasing replication efficiency in mammalian cells.

## Introduction

Influenza A virus (IAV) has two glycoproteins, hemagglutinin (HA) and neuraminidase (NA), on its enveloped surface, and these proteins play a crucial role in invasion and budding, respectively, in host cells. HA is a major protective antigen of IAV, and it has evolved to both escape host immune responses and adapt to new hosts via multiple amino acid substitutions (Matrosovich et al., 2000; Kim et al., 2013; Herve et al., 2015). Accumulation of additional N-glycosites near the receptor-binding site (RBS) of HA represents a significant evolutionary drift to evade pre-existing immunity (Hensley et al., 2009; Koel et al., 2013; Herve et al., 2015). During adaptation to terrestrial birds, the H5N1 avian influenza A virus (AIV) with additional N-glycans near the HA RBS balanced its relatively low HA binding affinity by decreasing its NA activity via NA stalk deletion (Matsuoka et al., 2009). These mutations facilitated the balance of HA-NA activities by increasing the replication efficiency and pathogenicity of AIVs in mammalian hosts (Matsuoka et al., 2009; Zhao et al., 2017).

To date, various glycoproteins and lectins in bodily fluids have been shown to inhibit the HA of IAVs (Gottschalk et al., 1972; Ng et al., 2012). Mucin is a glycoprotein, which can be secreted or cell-bound, in the mucus-producing epithelia of the respiratory, digestive, and reproductive tracts. It is also a highly glycosylated macromolecule containing abundant sialic acid residues linked to galactose by α2,3-linkages (Sia-α2,3-Gal), which function as a receptor for AIVs (Brayman et al., 2004; Adler et al., 2013). Ovomucin is present in egg white and is a strong HA inhibitor of AIVs; it is also absorbed and present in allantoic fluid (Lanni and Beard, 1948; Da Silva et al., 2017). The presence of IAV inhibitors in allantoic fluid has been previously reported (Svedmyr, 1949). The alpha 2 macroglobulin (α2M) and Ca2+-dependent (C-type) lectins bind mannose-rich N-glycans of HA and are important for the neutralization of IAVs (Anders et al., 1990; Matrosovich et al., 1998). The surfactant protein-D (SP-D) is another C-type lectin present in the lungs. It has been postulated that IAVs containing highly glycosylated HAs may be less pathogenic and have reduced replicative efficacy due to their increased susceptibility to C-type lectins (Matrosovich et al., 1998; Vigerust et al., 2007).

Based on previous reports, AIV A/chicken/01310/2000 (H9N2) (01310) was passaged 20 times in specific pathogen free (SPF)-embryonated chicken eggs (ECEs, 01310-CE20) to increase viral replication (Choi et al., 2008). In Korea, 01310-CE20 has been used as a vaccine strain in the inactivated oil emulsion vaccines. During passaging, 01310-CE20 has been shown to grow more efficiently in ECEs, causing more than 60% of embryonic deaths within 48 h (Choi et al., 2008). In addition, 01310-CE20 has been shown to acquire multiple mutations in coding genes, specifically three in HA (T133N, V216G, and E439D, H3 numbering) and two in NA (18 amino acid deletion (55–72) and E54D) (Choi et al., 2008). According to the mutation profiles, 01310-CE20 might have evolved a more effective balance of HA-NA activities through the generation of a new N-glycosite (T133N) in HA and a shortened stalk length in NA (Choi et al., 2008). In this study, we demonstrate significantly higher resistance of a 22-passage 01310 virus to innate inhibitors in egg white compared to a 4-passage 01310 virus. We further investigated the roles of egg-adapted mutations of HA and NA in innate inhibitor resistance and their correlation to mammalian pathogenicity.

## Materials and Methods

### Viruses, Eggs, and Cells

In this study, we used 01310-CE4 and 01310-CE22, which had been passaged 4 and 22 times, respectively, through the 10-day-old SPF ECEs (Charles River Laboratories, North Franklin, United States). The amino acid sequences of 01310-CE4 and 01310-CE22 were consistent with 01310-CE2 and 01310-CE20; therefore, we decided to refer to them as 01310-CE2 and 01310-CE20, respectively (**Supplementary Data S1**). To construct the plasmid DNA encoding the HA and NA segments of 01310-CE2 (H2 and N2) and 01310-CE20 (H20 and N20), a Hoffmann pHW2000 vector system was used as described previously (Hoffmann et al., 2000). Recombinant viruses were generated and passaged two times in 10-day-old SPF ECEs and then stored at −70°C until experimental use. Madin-Darby canine kidney (MDCK) and 293T cells were purchased from the Korean Collection for Type Cultures (KCTC, Korea) and maintained in DMEM (Life technologies Co., CA, United States) supplemented with 10% FBS (Life Technologies Co., CA, United States).

### Cloning and Rescue of Recombinant Viruses

Hemagglutinin and NA segments of 01310-CE2 and 01310-CE20 were cloned into the Hoffmann’s bi-directional transcription vector pHW2000, as described previously (Hoffmann et al., 2000). The nucleotide sequence of the insert was determined by sequencing with CMV-SF (5′-TAAGCAGAGCTCTCTGGCTA-3′) and bGH-SR (5′-TGGTGGCGTTTTTGGGGACA-3′) primers. Four combinations of HA and NA segments from 01310-CE2 and 01310-CE20 were mixed with six internal genome segments of IAV A/Puerto Rico/8/1934 (PR8) [300 ng of each plasmid + Lipofectamine 2000 + Plus reagents (Life Technologies Co., CA, United States)] and transfected into 293T cells as described previously, with modifications (Hoffmann et al., 2000; Kim et al., 2014). After an overnight incubation, 1 ml of Opti-MEM (Life Technologies Co., CA, United States) containing 1 μg/ml of L-1-tosylamido-2-phenylethyl chloromethyl ketone (TPCK)-treated trypsin (Sigma-Aldrich, MO, United States) were added to the transfected 293T cells. The culture medium was harvested after 24 h, and 200 μl of the medium was injected into 10-day-old SPF ECEs via the allantoic cavity. Three days after inoculation, the allantoic fluid was harvested and checked for virus growth by the HA assay, as recommended by the WHO manual on animal influenza diagnosis and surveillance. All mutant viruses were confirmed by RT-PCR and sequencing.

### Site-Directed Mutagenesis

To replace asparagine (N) with threonine (T) at position 133 (N133T), site-directed mutagenesis was performed using a Muta-direct Site Directed Mutagenesis Kit (iNtRON, Korea) as per the manufacturer’s protocol using the primers 01310-HA-N133T-F: 5′-CTTGGAATGTGACTTT CACTGGGACAAGCAAAGC-3′, and 5′-01310-HA-N133T-R: GCTTTGCTTGTCCCAGTGAA AGTCACATTCCAAG-3′. N133T was predicted to abolish a potential glycosylation site (133–135).

### N-Glycosite Prediction

To predict the three-dimensional 01310-CE20 HA protein structure, I-TASSER was used for homology modeling^1^ (Zhang, 2008). A glycan molecule was manually added to the predicted HA structure at position 133 using the Glyprot webserver^2^ (Bohne-Lang and von der Lieth, 2005).

### Deglycosylation Using PNGase F and Western Blotting

To confirm the presence of N-glycosylation at position 133 in 01310-CE20 HA, the recombinant viruses were denatured and deglycosylated with the PNGase F enzyme according to the manufacturer’s instructions (New England Biolabs, MA, United States). The viral proteins were separated by SDS–polyacrylamide gel electrophoresis (SDS–PAGE) using NuPAGE 4–12 % Bis-Tris Protein Gels (Life technologies Co., CA, United States); subsequently, they were transferred to a nitrocellulose membrane. The membrane was incubated with murine antisera induced by rH_20_N_20,_ followed by goat anti-mouse horseradish peroxidase (HRP)-conjugated secondary antibody (Abcam, Cambridge, United Kingdom). Protein bands were visualized using the Luminata forte western HRP substrate (Merck, Germany) and the ImageQuant LAS 4000 Mini (GE Healthcare Ltd., Buckinghamshire, United Kingdom).

### Hemagglutination Inhibition (HI) Assay

HI assay was performed according to the WHO manual for the laboratory diagnosis and virological surveillance of influenza, with modifications. Briefly, egg white, normal mouse lung extract, and mouse sera were serially diluted in 2-fold increments in 96-well plates, and four hemagglutinating units (HAU) of each virus were inoculated into each well. After 1 h of incubation at 4°C, 0.75 % guinea pig RBCs was added to each well. The hemagglutination inhibition (HI) titer was recorded after 1 h of incubation at 4°C. The HI titers are presented as the average of four independent experiments ±*SD*.

### Assay to Determine the Relative Replication Efficiency

To compare the MDCK cells infectivity relative to ECEs infectivity of the recombinant viruses, MDCK cells were seeded in 96-well plates at a density of 2×10^4^ cells/well. After 24 h, the confluent cells were washed twice with phosphate-buffered saline (PBS). Then, 10^7^ EID_50_/0.1 ml of the viruses were serially diluted from 10^−1^ to 10^−8^ in 10-fold increments, and 200 μl of each dilution was inoculated into each well with DMEM supplemented with 1 % bovine serum albumin (BSA) (fraction V) (Roche, Basel, Switzerland), 20 mM HEPES, antibiotic-antimycotic (Gibco, CA, United States), and 1 μg/ml TPCK-treated trypsin (Sigma-Aldrich, MO, United States). The TCID_50_ were measured at 3 and 5 days post-inoculation (dpi) by using HA test to determine the end point of virus growth, and the ratio of TCID_50_ to EID_50_ (TCID_50_/EID_50_) was calculated. The results were presented as an average of three independent experiments ± *SD*.

### Solid-Phase Assays of Receptor Binding Specificity

The receptor binding affinities of recombinant viruses were measured by a solid-phase binding assay as previously described by Matrosovich et al., with some modification (Matrosovich and Gambaryan, 2012). Briefly, 96-well enzyme linked immunosorbent assay (ELISA) plates (SPL, Korea) were coated with 10 μg/ml of fetuin (Sigma-Aldrich, MO, United States) and incubated overnight at 4°C. Once the plates were completely dry after washing, the recombinant viruses were bound to the fetuin-coated plates overnight at 4°C. Next, the wells were washed three times with PBS + 0.05 % Tween 20 and blocked with 0.1 % desialylated BSA + 10 μM of Oseltamivir (Sigma-Aldrich, MO, United States) for 1 h at 4°C. The washes were repeated three times, then the serially diluted biotinylated sialylglycopolymers (Neu5Acα2-3Galb1-4GlcNAcb-PAA-biotin, 3′SLN-PAA, and Neu5Acα2-6GalNAca-PAA-biotin, 6′SLN-PAA, Glycotech Corporation, MD, United States) were added to the plates and incubated for 1 h at 4°C. Finally, the plates were washed three times followed by incubation in horseradish peroxidase (HRP)-conjugated streptavidin (Thermo Fisher SCIENTIFIC, MA, United States) for 1 h at 4°C. The HRP was developed with the 3,3′,5,5′-Tetramethylbenzidine (TMB) substrate (SURMODICS, MN, United States), the reaction was stopped with 0.1 M H_2_SO_4_, and the absorbance at 450 nm was measured by a microplate reader (TECAN, Männedorf, Switzerland).

### Animal Experiments

Five-week-old (w-o) female BALB/c mice were purchased from KOATEC (Pyeongtaek, Korea), and all mouse experiments were performed at BioPOA Co. (Yongin, Korea). To measure the *in vivo* pathogenicity of each mutant virus, mice (*n* = 5) were anesthetized by intraperitoneal injection of 15 mg/kg Zoletil 50 (Virbac, Carros, France). Anesthetized mice were inoculated intranasally with 10^6^ EID_50_/50 μl of each virus. The negative control (Mock) mice were injected with the same volume of sterilized PBS. Mortality and weight loss were measured for 10 days. Mice that lost more than 20% of their original weight were euthanized and recorded as a death. Anti-rH_20_N_20_ serum was collected from the mice that survived rH_20_N_20_ viral infection and was used for Western blot analysis. For comparing viral replication efficiency in the lungs of infected mice, four mice from each group were injected with PBS (Mock) or 10^6^ EID_50_/50 μl of each of the recombinant virus. Mouse lungs were collected at 3 dpi and stored at −70°C until experimental use. Tissues were ground using a TissueLyser 2 (Qiagen, Valencia, CA, United States) with 5-mm stainless-steel beads and 100 μl of PBS in suspension. Then, PBS was added to make a 10 % suspension of the ground tissues. After centrifugation at 2000 ×*g* for 10 min, the supernatant was harvested, and virus titers of the lung homogenates were measured based on the TCID_50_ per lung.

### Ethics Statement

All mouse experiments were performed at BioPOA Co. (Yongin, Korea) following a protocol that adhered to the National Institutes of Health’s public health service policy on the humane care and use of laboratory animals. The protocol was reviewed and approved by the Institutional Animal Care and Use Committee (IACUC) of BioPOA Co. (BP-2016-005-2).

## Results

### Comparison of Resistance to Innate Inhibitors in Egg White Between 01310-CE2 and 01310-CE20

**FIGURE 1:**
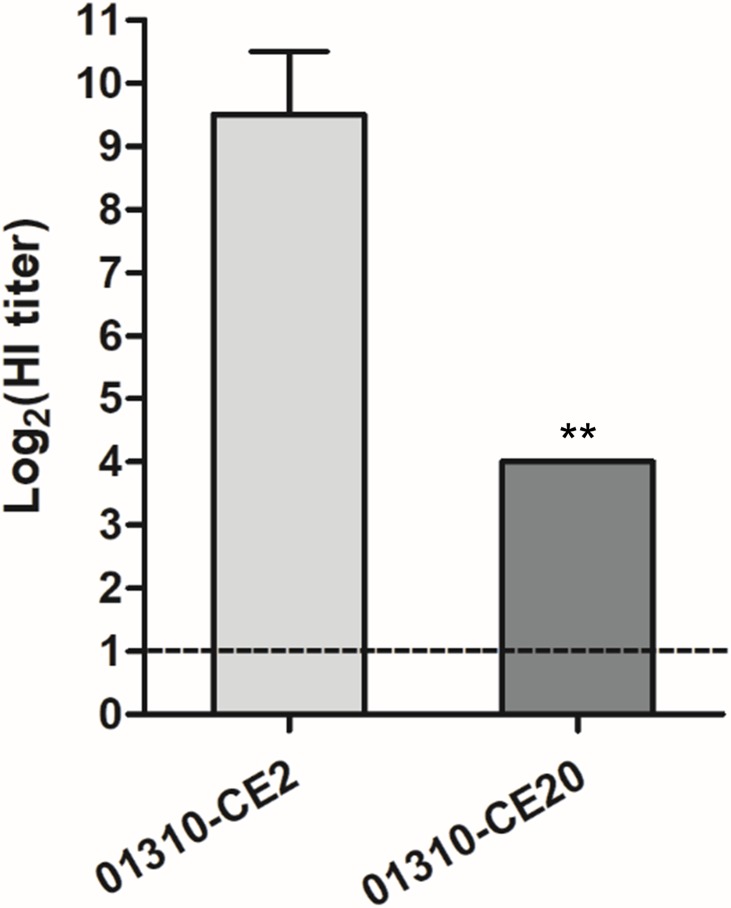
Comparison of resistance to egg white between 01310-CE2 and 01310-CE20 viruses. Egg white is serially diluted in 2-fold increments in 96-well plates, and four HAUs of each virus were inoculated into each well. Subsequently, the plates were incubated for 1 h, and 0.75 % guinea pig RBCs were added. The hemagglutination inhibition levels were measured after an additional 1 h of incubation. The data represents the average of four independent experiments ± *SD*. Statistical significance was analyzed using a Student’s *t*-test (^∗∗^*P* < 0.01).

It has been previously reported that innate inhibitors prevent hemagglutination of IAVs in normal allantoic fluid (Svedmyr, 1949), and we hypothesized that these inhibitors might influence the selection pressure during egg adaptation of AIVs. To verify this hypothesis, we compared the HI titers of 01310-CE2 and 01310-CE20 viruses in the presence of egg white. Egg white inhibited the HA of 01310-CE2 approximately 32 times more efficiently than 01310-CE20 (**Figure 1**). Therefore, 01310-CE20 became more resistant to innate inhibitors during egg adaptation.

### Comparison of the Relative MDCK Cell Infectivity

**FIGURE 2:**
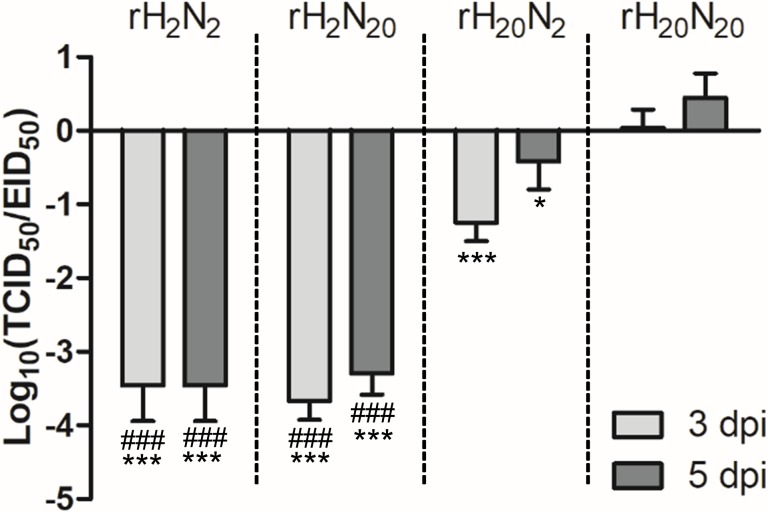
The relative replication efficiency of recombinant viruses in MDCK cells to ECEs. MDCK cells were infected with 10^7^ EID_50_/0.1 ml of the recombinant viruses, and the TCID_50_/EID_50_ ratio was determined at 3 and 5 dpi. The data represent the average of three independent experiments ±*SD*. Statistical significance was analyzed by one-way ANOVA (compared to rH_20_N_20_, ^∗^*P* < 0.05, ^∗∗∗^*P* < 0.001, compared to rH_20_N_2_, ^###^*P* < 0.001).

Given the amino acid substitutions in 01310-CE20, we hypothesized that its HA and/or NA genes may drive the increased resistance to innate inhibitors. Therefore, we generated four PR8-derived recombinant viruses with different combinations of HA and NA genes from 01310-CE2 and 01310-CE20 (rH_2_N_2_, rH_2_N_20_, rH_20_N_2_, and rH_20_N_20_) and compared their relative MDCK cell infectivity at 3 and 5 dpi (**Figure 2**). rH_20_N_2_ and rH_20_N_20_, which contain the HA genes of 01310-CE20, had a higher MDCK cell/ECEs infectivity ratio than rH_2_N_2_ and rH_2_N_20_, which contain the HA genes of 01310-CE2 (**Figure 2**). The replication efficiency of rH_2_N_20_ was not different from that of rH_2_N_2_, but rH_20_N_20_ had a higher MDCK cell/ECEs infectivity ratio than rH_20_N_2_ (**Figure 2**). This finding suggests that both HA and NA of 01310-CE20 play important roles in viral replication in MDCK cells, but HA’s role may be more crucial for viral replication.

### Comparison of the Pathogenicity of Recombinant Viruses in BALB/c Mice

**FIGURE 3:**
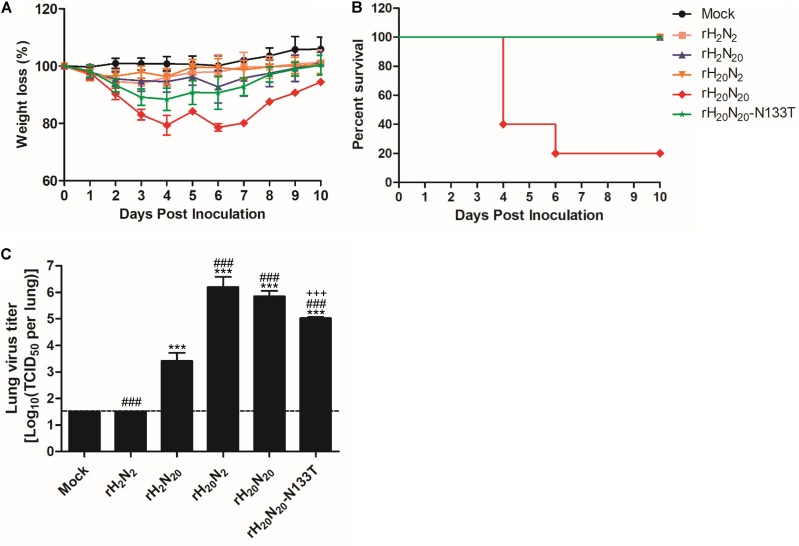
Pathogenicity of the recombinant viruses in mice. The virulence of the recombinant viruses was determined based on **(A)** body weight loss and **(B)** the mortality of infected mice. Five 5-week-old BALB/c mice were challenged with 1.0 × 10^6^ EID_50_ of each virus or PBS (Mock). Mortality and weight loss were observed for 10 days. Average weight loss ± SD was measured relative to the initial weight of each mouse. **(C)** Viral replication in the mouse lung at 3 dpi. The three mice infected with 1.0 × 10^6^ EID_50_ of each virus were sacrificed at 3 dpi, and the viral titers were measured by TCID_50_ per lung. Statistical significance was analyzed by one-way ANOVA (compared to rH_2_N_2_, ^∗∗∗^*P* < 0.001, compared to rH_2_N_20_,^###^*P* < 0.001, compared to rH_20_N_20_, ^+++^*P* < 0.001).

To investigate the roles of HA and NA genes from 01310-CE20 in mammalian pathogenicity, we injected the recombinant viruses into BALB/c mice intranasally and observed the resulting mortality and morbidity. rH_20_N_20_ infection led to significant weight loss in BALB/c mice, and eventually resulting in euthanization of 4 out of 5 inoculated mice at days 4 and 6 (**Figures 3A,B**). However, the other recombinant viruses did not cause death in BALB/c mice. In contrast to rH_2_N_2_, rH_2_N_20_ replicated in the mouse lung at 3 dpi. Further, both rH_20_N_20_ and rH_20_N_2_ viruses showed significantly higher replication efficiencies in the mouse lung than rH_2_N_2_ and rH_2_N_20_ (*p* < 0.05) (**Figure 3C**). Thus, the HA and NA genes of 01310-CE20 could individually increase viral replication in the murine lungs, but not enough to cause body weight loss and mortality in BALB/c mice. However, if these mutations were combined, the virus could obtain sufficient pathogenicity to cause body weight loss and mortality in BALB/c mice.

### Different Resistances of Recombinant Viruses to Innate Inhibitors in Egg White, Mouse Sera, and Mouse Lung Extracts

**FIGURE 4:**
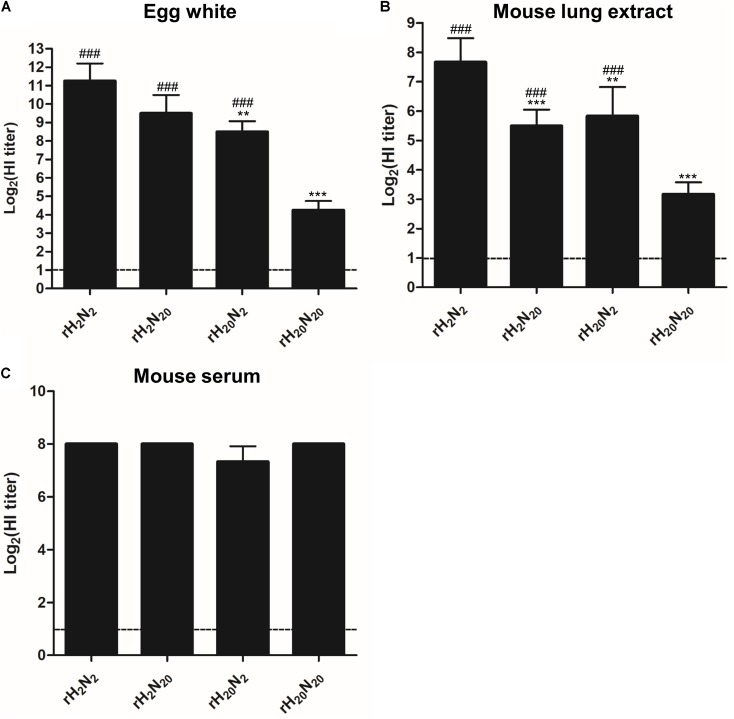
Comparison of resistance to innate inhibitors. The resistance of the recombinant viruses to **(A)** egg white, **(B)** mouse lung extract, and **(C)** mouse sera were measured by the hemagglutination inhibition assay. The innate inhibitors were serially diluted in 2-fold increments in 96-well plates, and four HAUs of each virus were inoculated into each well. The data represent the average of four independent experiments ± *SD*. Statistical significance was analyzed by one-way ANOVA (compared to rH_2_N_2_, ^∗∗^*P* < 0.01, ^∗∗∗^*P* < 0.001, compared to rH_20_N_20_,^###^*P* < 0.001).

To evaluate the effects of HA and NA genes from 01310-CE20 on innate inhibitor resistance, we compared the HI titers of egg white, normal mouse lung extracts, and mouse sera against different recombinant viruses. The HI titers from egg whites for both rH_20_N_2_ and rH_20_N_20_ were significantly lower than those of rH_2_N_2_, although rH_20_N_20_ was less inhibited by egg white than rH_20_N_2_ (**Figure 4A**). Similarly, the HI titers from lung extracts for rH_2_N_20_, rH_20_N_2_, and rH_20_N_20_ were significantly lower than those of rH_2_N_2_, and the HI titers for rH_20_N_20_ were significantly lower than those for rH_20_N_2_ (**Figure 4B**). However, the HI titers from the serum of all the recombinant viruses were relatively high and did not show significant differences from each other (**Figure 4C**). These findings suggest that HA and NA from 01310-CE20 may play common roles in establishing resistance to innate inhibitors of both avian and mammalian hosts. Meanwhile, HI assays using recombinant human SP-D (Sino biological Inc., China) showed that all four recombinant viruses were not inhibited by 125 μg/ml of SP-D (**Supplementary Figure S1**).

### Glycosylation Pattern of Recombinant Viral Hemagglutinin

**FIGURE 5:**
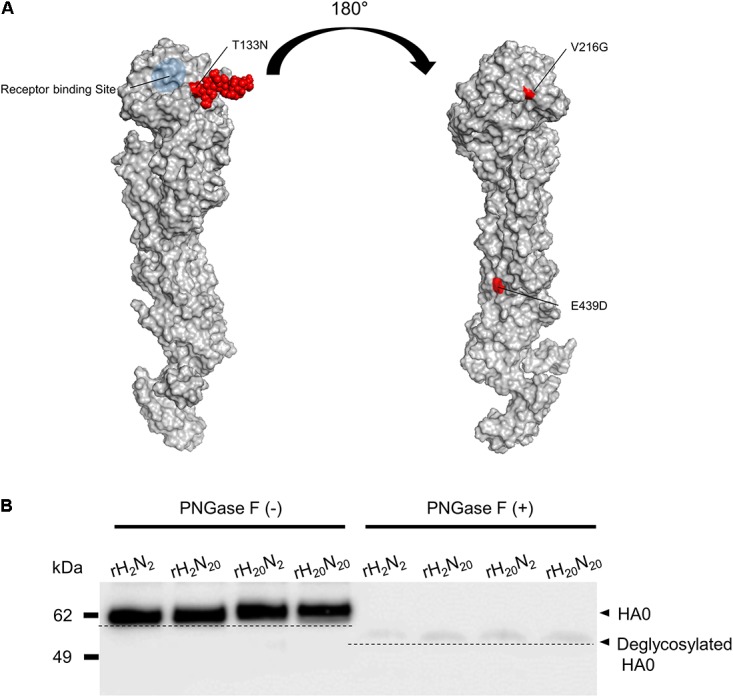
Additional N-glycosylation of the 01310-CE20 HA protein. **(A)** Structural model of the 01310-CE20 HA protein. The predicted structural model was derived from homology modeling with I-TASSER, and a glycan molecule (*red*) was manually added at position 133 using the Glyprot webserver. **(B)** Different molecular weights of HA1 protein due to additional N-glycosylation at position 133. Western blot analysis was performed using rH_20_N_20_ antisera as the primary antibody, and the blots were visualized using an ImageQuant LAS 4000 Mini.

Of the HA mutations acquired by 01310-CE20, the T133N mutation occurred in the vicinity of the RBS and generated a new potential N-glycosylation site at position N133 (**Figure 5A**). To determine whether N-glycosylation occurred in this region, we compared the molecular weights of the HA0 protein from each of the recombinant viruses using Western blot analysis (**Figure 5B**). We found that the molecular weights of rH_20_N_2_ and rH_20_N_20_ HA0 proteins (unlike their PNGase F-treated counterparts) were slightly higher than those of rH_2_N_2_ and rH_2_N_20_, consistent with an additional glycosyl moiety (**Figure 5B**). Considering the point mutations acquired in 01310- CE20 HA1 (T133N, V216G), these results are consistent with the presence of an additional N-glycosyl moiety at N133. Meanwhile, the weak signal of the PNGase F-treated counterparts suggest that the deglycosylation may affect the recognition of the HA protein by altering the HA antigenicity (Zost et al., 2017; Mostafa and Pleschka, 2018).

### Loss-of-Function (LOF) Effects of N133T Mutation on Virus Replication, Mammalian Pathogenicity, and Resistance to Innate Inhibitors

**FIGURE 6:**
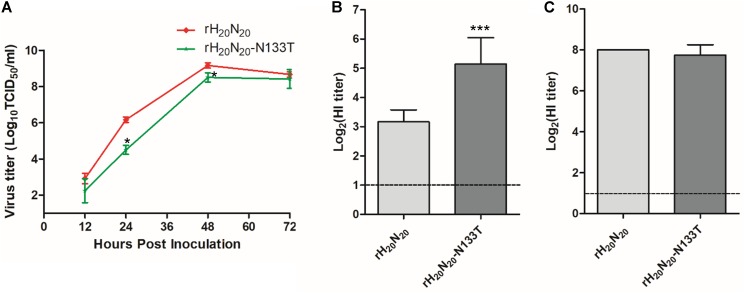
Effects of N133T mutation on viral replication and resistance to innate inhibitors. **(A)** Growth kinetics of rH_20_N_20_ and rH_20_N_20_-N133T in MDCK cells. MDCK cells were infected with 0.001 MOI of each virus. At 12, 24, 48, and 72 h post-inoculation, the viral titers in the supernatants were measured using TCID_50_. The resistance of rH_20_N_20_-N133T in **(B)** mouse lung extracts and **(C)** mouse sera was measured by the hemagglutination inhibition assay. The data represent the average of four independent experiments ± *SD*. Statistical significance was analyzed using one-way ANOVA (compared to rH_20_N_20_, ^∗^*P* < 0.05, ^∗∗∗^*P* < 0.001).

To investigate the effects of T133N mutation on virus replication efficiency, mammalian pathogenicity, and innate inhibitor resistance, we generated a LOF mutant virus possessing an N133T mutation in HA, rH_20_N_20_-N133T, and compared its traits with those of rH_20_N_20_. The viral titer of rH_20_N_20_-N133T in MDCK cells was significantly lower than that of rH_20_N_20_ at 24 and 48 h post-inoculation (**Figure 6A**). rH_20_N_20_ caused significant body weight loss and mortality in inoculated mice, but rH_20_N_20_-N133T, similar to rH_2_N_20_, only caused body weight loss, with no mortality (**Figures 3A,B**). Furthermore, in comparison to rH_20_N_20_, rH_20_N_20_-N133T showed significantly decreased lung viral titers in mice (**Figure 3C**). It also exhibited lower resistance to innate inhibitors in the mouse lung than rH_20_N_20_ (**Figure 6B**). However, the HI titers from the mouse serum of rH_20_N_20_-N133T did not show any differences (**Figure 6C**). Thus, the LOF mutation (N133T) decreased viral replication efficiency in MDCK cells and mouse lungs and decreased *in vivo* pathogenicity and resistance to innate inhibitors.

### The Effect of Different HA and NA Mutations on the Receptor Binding Affinities to Avian-Like and Human-Like Receptors

**FIGURE 7:**
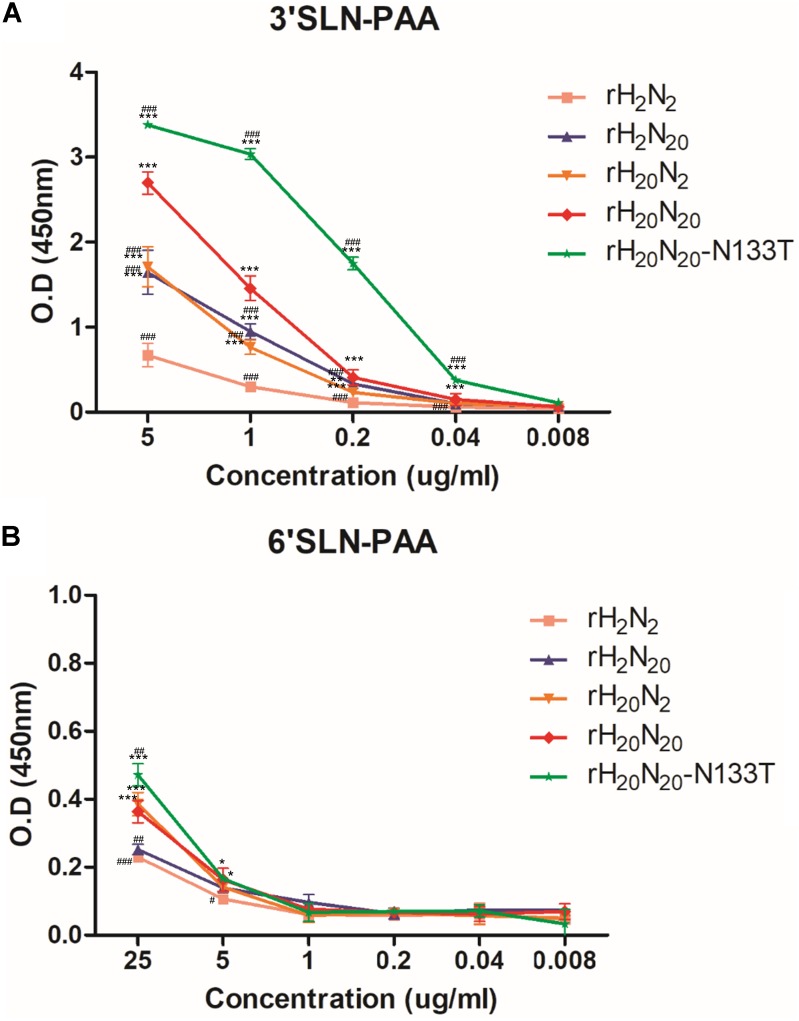
Comparison of receptor binding affinities to human- and avian-like receptors. Receptor binding avidities of recombinant viruses were measured using the solid-phase direct binding assay. The recombinant viruses were bound to fetuin-coated plates. Subsequently, serially diluted biotinylated avian-like receptor sialylglycopolymers (Neu5Acα2-3Galb1-4GlcNAcb-PAA-biotin [3′SLN-PAA]) and human-like receptor sialylglycopolymers (Neu5Acα2-6GalNAca-PAA-biotin [6′SLN-PAA]) were added. The plates were developed using an HRP-conjugated streptavidin, TMB substrate solution, and stop solution; the absorbance at 450 nm was measured by a microplate reader. **(A)** Receptor binding avidities of recombinant viruses to avian-like receptor. **(B)** Receptor binding avidities of recombinant viruses to human-like receptor. The data represents the average of three independent experiments ±*SD*. Statistical significance was analyzed by one-way ANOVA (compared to rH_2_N_2_, ^∗^*P* < 0.05, ^∗∗^*P* < 0.01, ^∗∗∗^*P* < 0.001; compared to rH_20_N_20_,^#^*P* < 0.05, ^##^*P* < 0.01, ^###^*P* < 0.001).

The receptor binding affinity was measured by solid-phase direct binding assays (Matrosovich and Gambaryan, 2012). Among the four recombinant viruses (rH_2_N_2_, rH_2_N_20_, rH_20_N_2_, and rH_20_N_20_), rH_2_N_2_ showed the lowest receptor binding affinity to 3′SLN-PAA (Sia-α2,3-Gal, avian-like receptor) (**Figure 7A**). rH_2_N_20_ and rH_20_N_2_ exhibited moderate affinities, and rH_20_N_20_ exhibited the highest receptor binding affinity to the avian-like receptor (**Figure 7A**). These findings demonstrate that egg-adapted HA and NA may collaboratively contribute to an increased binding affinity to the avian-like receptor (Gambaryan et al., 1999). Although all four recombinant viruses showed generally lower binding affinities to 6′SLN-PAA (Sia-α2,6-Gal, mammalian-like receptor) than to the avian-like receptor, it was confirmed that the binding affinities were increased by introducing the HA gene of 01310-CE20 viruses (rH_20_N_2_ and rH_20_N_20_) (**Figure 7B**). Therefore, egg-adapted HA gene is involved in the receptor binding affinity to both avian-like and human-like receptors. Interestingly, rH_20_N_20_-N133T also showed significantly higher binding affinities to both avian-like (**Figure 7A**) and human-like (**Figure 7B**) receptors than rH_20_N_20_. Thus, the additional N-glycosylation in 01310-CE20 HA decreased viral receptor binding affinities in both avian-like and human-like receptors.

## Discussion

There are innate inhibitors hindering the hemagglutination of AIVs in normal chicken allantoic fluid. Ovomucin, which is abundant in egg white, is likely to play a crucial role as an innate inhibitor based on its similar binding patterns with allantoic fluid to egg-adapted IAVs (Gambaryan et al., 1999; Da Silva et al., 2017). Egg adaptation of human IAVs results in increased affinity to avian-like receptor and/or escape from binding to inhibitors in the allantoic fluid (Hardy et al., 1995; Gambaryan et al., 1997, 1999). Given that the chicken may be a potential intermediate host to force H9N2 avian influenza viruses to acquire the mammalian adaptive markers (Wan and Perez, 2007; Kuchipudi et al., 2009; Chrzastek et al., 2018), egg adaptive mutations of avian IAVs may affect viral pathogenicity in mammals. The recombinant viruses containing the 01310-CE20 HA (rH_20_N_2_ and rH_20_N_20_) displayed increased binding affinities to both avian-like and mammalian-like receptors, and increased resistance to the innate inhibitors in egg white and mouse lungs. Similar to innate inhibitors inducing egg-adapted mutations, antigenic drift was also demonstrated through antibody targeting of the 2009 pandemic H1N1 virus HA, which resulted in an increased virulence in mice and altered their receptor-binding properties (O’Donnell et al., 2012). Taken together, these adaptive mutations may aid IAVs to escape antibody- or inhibitor-based immunological pressures by increasing receptor-binding affinities and virulence.

Recombinant viruses containing the 01310-CE20 HA (rH_20_N_2_ and rH_20_N_20_) displayed higher replication efficiency in MDCK cells than those containing the 01310-CE2 HA (rH_2_N_2_, rH_2_N_20_). The viral replication of the avian-origin recombinant viruses in MDCK cells may not be restricted because of the existence of both avian-like and mammalian-like receptors on the MDCK cells (Ito et al., 1997). The relatively higher replication capacities of rH_20_N_2_ and rH_20_N_20_ can be explained by the increased affinities to both receptors on MDCK cells. Moreover, the significantly higher replication efficiency of rH_20_N_20_ than rH_20_N_2_ may be related to increased affinity to avian-like receptor. MDCK cells are known to secrete apically small glycoproteins along with the mammalian-like receptor, and this may impact growth of IAVs (Ohkura et al., 2002). Stalk length of NA does not influence NA activity for small substrates, but the enzyme activity of avian NA to cleave the mammalian receptor was lower than that of mammalian NA (Els et al., 1985; Castrucci and Kawaoka, 1993; Kobasa et al., 1999; Garcia et al., 2014). The LOF mutant rH_20_N_20_-N133T showed higher affinities to avian-like and mammalian-like receptors but significantly lower replication efficiency in MDCK cells than rH_20_N_20_ in the period 24–48 h post-inoculation. The loss of N-glycosylation at position 158 in H5N1 HA also increased the affinity to human-like receptor (Stevens et al., 2008; Wang et al., 2010). Similar to H5N1 viruses, the deglycosylation of rH_20_N_20_ may increase the affinity to sialic acid receptors. Therefore, the decreased replication efficiency of rH_20_N_20_-N133T may be associated with its increased affinity to mammalian-like receptor on apically secreted glycoproteins from MDCK cells.

The most common animal model, the BALB/c mouse, expresses both avian-like and mammalian-like receptors in the lower respiratory tract (Ning et al., 2009). The replication efficiencies of rH_20_N_2_ and rH_20_N_20_ in the mouse lung were similar but significantly higher than those of rH_2_N_2_ and rH_2_N_20_. In addition, rH_20_N_20_-N133T showed lower replication efficiency in the mouse lung than rH_20_N_20_, but higher than rH_2_N_2_ and rH_2_N_20_. The higher affinity of rH_20_N_20_ to avian-like receptor than rH_20_N_2_ might explain the rH_20_N_20_-associated deaths in mice. However, considering the higher affinity of rH_20_N_20_-N133T to avian-like receptors than rH_20_N_20_, the resistance to innate inhibitor may also play an important role in the viral pathogenicity in mice. In the murine respiratory tract, two well-known innate inhibitors, mucin and SP-D, are secreted (Lamblin and Roussel, 1993; Hartshorn et al., 1994; Barbier et al., 2012; Ng et al., 2012; Zanin et al., 2016). Whereas mucin can bind to either the RBS of HA or the hemadsorption site of NA, SP-D binds to glycans present in HA and NA (Lamblin and Roussel, 1993; Reading et al., 1997; Zanin et al., 2016). The sensitivity of N-glycans to neutralization by SP-D are known to vary; in particular, specific N-glycans, such as the 130N-glycan of the H1 IAVs and 165N-glycan of H3 IAVs, can determine this sensitivity (Job et al., 2010; Tate et al., 2011a,b). In this study, we demonstrated that the egg-adapted HA of 01310-CE20 increased viral resistance to innate inhibitors with no difference in viral resistance to SP-D. Further, we demonstrated that the N-glycosite at position 133 decreased viral binding affinities to both avian-like and mammalian-like receptors coupled with increased resistance to innate inhibitors. Therefore, the 133N-glycan near the RBS of HA1 may be more involved in the resistance to mucin than SP-D.

V216G and E439D mutations in HA may not be present in the vicinity of the receptor binding site (**Figure 5**). However, considering the higher virus titer of rH_20_N_20_-N133T (with V216G and E439D mutations) than rH_2_N_20_ (without V216G and E439D mutations) in the lungs of infected mice, these mutations may influence their replication in mice (**Figure 3C**). Meanwhile, the significantly different innate inhibitor resistance observed between recombinant viruses with different neuraminidases (e.g., rH_2_N_2_ vs. rH_2_N_20_, and rH_20_N_2_ vs. rH_20_N_20_) may indicate the importance of NA in this resistance. The optimal balance of HA-NA activities is one of the important factors forcing the AIVs to become transmissible into mammals (Wagner et al., 2000; Baigent and McCauley, 2001; Matsuoka et al., 2009). The combination of HA and NA from 01310-CE20, naturally arising in the course of adaptation, led to the highest infectivity and virulence in mice. Although we do not have data on which mutations in HA and NA occurred first, the T133N mutations located in the vicinity of RBS in HA1 and the 18 amino acid deletion of the NA stalk of 01310-CE20 may be the best combination for viral fitness, balancing optimal affinities to receptors on host cells and innate inhibitors (Gambaryan et al., 1999; Wagner et al., 2000; Lu et al., 2005; Li et al., 2011).

Previously, 01310-CE20 showed increased pathogenicity, causing early embryonic death after ECEs inoculation (Choi et al., 2008). Our findings demonstrate that this increased pathogenicity in ECEs is deeply involved in not only increased viral replication, but also increased viral resistance to innate inhibitors. However, the lack of early embryonic death after inoculation of rH_20_N_2_ and rH_20_N_20_ with six PR8-derived internal genes may reflect the importance of multigenic traits and internal genes for the embryonic pathogenicity of AIVs (Song et al., 2008).

In this study, we indicate that egg adaptation of a H9N2 AIV, 01310-CE20, is the result of mutations balancing the affinities of HA and NA to both avian-like and human-like receptors on host cells and innate inhibitors. Therefore, AIVs acquire common, essential mutations necessary for efficient replication in mammalian hosts during adaptation in another intermediate host, the chicken, and these mutations may be essential to acquire additional mammalian pathogenicity-related mutations (Lee et al., 2017).

## Author Contributions

H-JK and C-YL designed the study, analyzed the data, and wrote the manuscript. C-YL and S-HA performed the experiments. J-HK, Y-JL, and J-GC contributed to data interpretation. Y-JL and J-GC provided the 01310 viruses and genetic information.

## Conflict of Interest Statement

The authors declare that the research was conducted in the absence of any commercial or financial relationships that could be construed as a potential conflict of interest.

## Footnotes

1 https://zhanglab.ccmb.med.umich.edu/I-TASSER/

2 http://www.glycosciences.de/modeling/glyprot/php/main.php

## Supplementary Material

FIGURE S1 Comparison of resistance to SP-D. For recombinant viruses, their resistance to recombinant human SP-D (125 μg/ml) was measured by the hemagglutination inhibition assay. SP-D was serially diluted in 2-fold increments in 96-well plates, and four HAUs of the viruses were inoculated into each well. The data represent the average of three independent experiments.

DATA S1 The nucleotide sequences of 01310 viruses which had been passaged 2, 4, 20, and 22 times through 10-day-old SPF ECEs.
