# Supplementary material for: Acquisition of Innate Inhibitor Resistance and Mammalian Pathogenicity During Egg Adaptation by the H9N2 Avian Influenza Virus
Source: Front Microbiol. 2018 Aug 21;9:1939. doi: 10.3389/fmicb.2018.01939 (PMC6110911; doi:10.3389/fmicb.2018.01939)
Supplement: FIGURE S1 — Comparison of resistance to SP-D. For recombinant viruses, their resistance to recombinant human SP-D (125 μg/ml) was measured by the hemagglutination inhibition assay. SP-D was serially diluted in 2-fold increments in 96-well plates, and four HAUs of the viruses were inoculated into each well. The data represent the average of three independent experiments. [file Image_1.PDF]

Supplementary Material

Acquisition of innate inhibitor resistance and mammalian pathogenicity during egg adaptation by an H9N2 avian influenza virus

Chung-Young Lee, Se-Hee An, Jun-Gu Choi, Youn-Jeong Lee, Jae-Hong Kim, Hyuk-Joon Kwon*

*** Correspondence:** Hyuk-Joon Kwon: kwonhj01@snu.ac.kr

1 Supplementary Data

Supplementary Data 1. The nucleotide sequences of 01310 viruses which had been passaged 2, 4, 20, and 22 times through 10-day-old SPF ECEs.

2 Supplementary Figures and Tables

2.1 Supplementary Figures

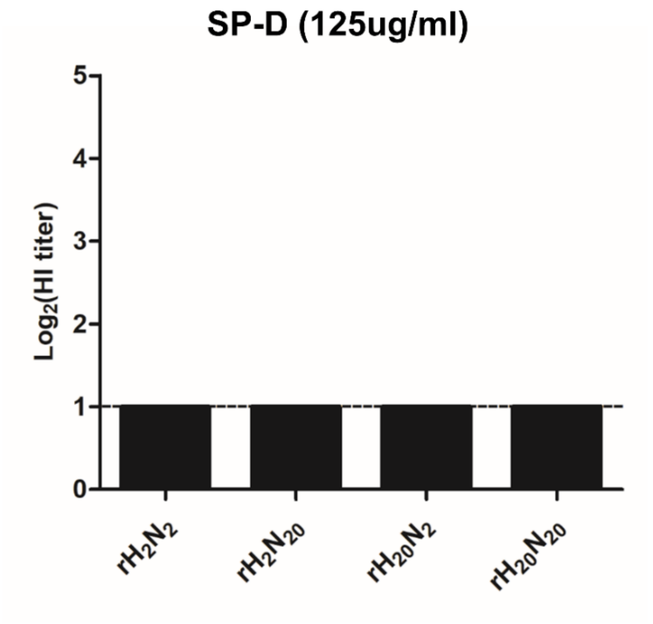

Supplementary Figure 1. Comparison of resistance to SP-D. For recombinant viruses, their resistance to recombinant human SP-D (125 µg/ml) was measured by the hemagglutination inhibition assay. SP-D was serially diluted in 2-fold increments in 96-well plates, and four HAUs of the viruses were inoculated into each well. The data represent the average of three independent experiments.
